# ARF6-Mediated Endosome Recycling Reverses Lipid Accumulation Defects in Niemann-Pick Type C Disease

**DOI:** 10.1371/journal.pone.0005193

**Published:** 2009-04-14

**Authors:** Jill Kuglin Schweitzer, Sean D. Pietrini, Crislyn D'Souza-Schorey

**Affiliations:** Department of Biological Sciences and the Center for Rare and Neglected Diseases, University of Notre Dame, Notre Dame, Indiana, United States of America; University of Geveva, Switzerland

## Abstract

In human Niemann-Pick Type C (NPC) disease, endosomal trafficking defects lead to an accumulation of free cholesterol and other lipids in late endosome/lysosome (LE/LY) compartments, a subsequent block in cholesterol esterification and significantly reduced cholesterol efflux out of the cell. Here we report that nucleotide cycling or cellular knockdown of the small GTP-binding protein, ARF6, markedly impacts cholesterol homeostasis. Unregulated ARF6 activation attenuates the NPC phenotype at least in part by decreasing cholesterol accumulation and restoring normal sphingolipid trafficking. These effects depend on ARF6-stimulated cholesterol efflux out of the endosomal recycling compartment, a major cell repository for free cholesterol. We also show that fibroblasts derived from different NPC patients displayed varying levels of ARF6 that is GTP-bound, which correlate with their response to sustained ARF6 activation. These studies support emerging evidence that early endocytic defects impact NPC disease and suggest that such heterogeneity in NPC disease could result in diverse responses to therapeutic interventions aimed at modulating the trafficking of lipids.

## Introduction

Cholesterol enters the endosomal recycling compartment (ERC), a major repository of free cholesterol, via endocytosis and non-vesicular mechanisms [1]. Cells acquire exogenous cholesterol primarily through LDL-mediated endocytosis. The LDL particle transits along the endosomal network to the LE/LY, where it is hydrolyzed by acid lipase. Then, the free cholesterol moves to the ER or returns to the PM via vesicular and non-vesicular transport [1]. In addition, LDL-derived free cholesterol may move to the plasma membrane without transiting to LE/LY since acid lipase is also present in early endocytic organelles [2]. NPC arises from mutations in either NPC1 or NPC2, proteins of unknown function that localize to the LE/LY at steady state and are believed to play a role in cholesterol and/or lipid transport [3]. In NPC cells, LDL-cholesterol as well as endogenous cholesterol and other lipids accumulate aberrantly in LE/LY compartments and efflux of cholesterol, particularly LDL-derived cholesterol, is greatly diminished [2], [4]–[7]. Defects in membrane trafficking accompany the NPC phenotype. In particular, cells lacking NPC1 exhibit reduced recycling of lipid, protein, and fluid-phase markers to the PM [2], [8], [9]. Since cholesterol homeostasis relies, in part, on membrane recycling, an underlying problem in NPC may be the inefficient recycling of cholesterol and other lipids. Thus, modulation of this pathway represents a potential target for correcting the aberrant cholesterol accumulation in the LE/LY that is a hallmark of NPC disease.

ARF6 is a small GTPase that regulates the trafficking of endosomal membrane [10]. Here we tested the hypothesis that ARF6 activation enhances cholesterol efflux via its effect on early endosome recycling, thereby reducing cholesterol accumulation observed in NPC. We find that constitutively active ARF6 reverts the NPC phenotype in part, including a decrease in filipin intensity, an increase in cholesterol efflux, and a restoration of normal sphingolipid trafficking. We also show that a concomitant decrease in the amount of endogenous ARF6 that is GTP-bound accompanies cholesterol storage in some NPC fibroblasts. Furthermore, we show that the observed decrease in cholesterol accumulation in NPC fibroblasts upon sustained ARF6 activation correlates with their relative level of endogenous ARF6-GTP. These studies support a role for ARF6 in lipid trafficking and suggest that sustained stimulation of endosomal recycling increases cholesterol efflux out of the cell, thereby decreasing intracellular cholesterol accumulation.

## Results and Discussion

**Figure 1:**
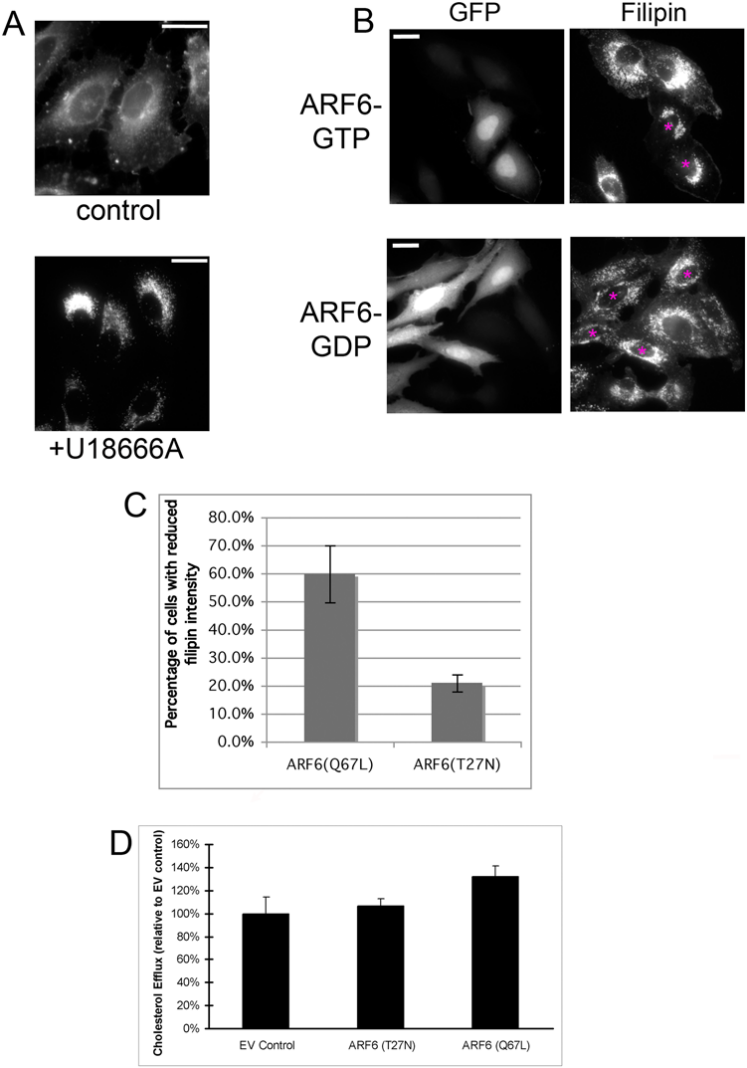
Constitutively active ARF6 increases cholesterol removal in NPC-like cells. *A*, HeLa cells (with or without 1 µg/ml U18666A treatment for 24 h) were fixed and stained with filipin. Note cholesterol accumulation in cells treated with U18666A. Bars, 20 µm. *B*, HeLa cells were treated with U18666A to induce cholesterol accumulation and then transfected with pIRES-GFP encoding ARF6(Q67L), the ARF6-GTP mutant, or ARF6(T27N), the ARF6-GDP mutant, and fixed approximately 24 h post-transfection. Left panels show GFP expression and right panels show filipin staining. Transfected cells are marked with asterisks in filipin images. Bars, 20 µm. *C*, Quantitation of the percentage of transfected cells with reduced filipin intensity (see Methods). For each condition, the average of three independent experiments is shown with standard error bars. The difference between control cells and ARF6(Q67L)-expressing cells is statistically significant (p = 0.021), using a two-tailed t-test. *D,* Relative cholesterol efflux from HeLa cells treated with U18666A and then transfected with pIRES-GFP (EV) or pIRES-GFP encoding ARF6(Q67L) or ARF6(T27N). The average of three independent experiments is shown with standard error bars. Statistically significant comparisons: ARF6(Q67L) vs. EV, p = 0.024 and ARF6(Q67L) vs. ARF6(T27N), p = 0.037. The actual percentage of cellular cholesterol effluxed in each case: 2.41% for EV control, 2.56% for ARF6(T27N), and 3.18% for ARF6(Q67L).

Previous results suggest that cholesterol cycles, in part, through vesicles containing the GTP-binding protein, ARF6, a potent regulator of early endosomal membrane internalization and recycling [10], [11]. Consistent with these findings, we find significant overlap of cholesterol with ARF6 in HeLa cells (Figure S1A). To mimic abnormal cholesterol accumulation observed in a variety of lipid storage diseases, we treated HeLa cells with the hydrophobic polyamine U18666A. Treatment with U18666A induces acute cholesterol accumulation that is indistinguishable from that seen in NPC (Figure 1A) [12]. U18666A blocks cholesterol exit from LE/LY likely by binding negatively charged phospholipids in late endosomal membranes, such as LBPA [13], [14]. Since ARF6 activation is coupled to the efflux of recycling endosomal membrane [10], we examined the impact of constitutive ARF6 activation on the lipid accumulation phenotype induced by U18666A. To this end, HeLa cells were transfected with a GTPase defective, constitutively activated ARF6 mutant, ARF6(Q67L), after incubation with U18666A for 6–8 h to induce cholesterol accumulation. Cells were stained with filipin to visualize cholesterol. Sustained ARF6 activation induced a marked decrease in filipin staining compared to non-transfected cells in the same experiment (Figure 1B, top row, and Figure S1B). To quantitate the reduction in cholesterol accumulation induced by sustained ARF6 activation, we measured the filipin intensity in transfected and untransfected cells and found that approximately 60% of the cells expressing exogenous ARF6-GTP exhibited reduced filipin intensity compared to non-transfected cells (Figure 1C). In contrast, when cells were transfected with ARF6(T27N), the dominant negative ARF6 mutant, no significant change in filipin intensity was observed compared to untransfected cells (Figure 1B, bottom row, and 1C).

Our results suggest that sustained activation of ARF6 induces exit of cholesterol from LE/LY compartments. We propose that cholesterol “trapped” in the LE/LY is accessible for removal in response to modulation of upstream steps along the endosomal pathway, in our case endosomal recycling. To test this hypothesis, we determined whether sustained ARF6 activation promotes cholesterol removal from U18666A-treated cells and measured ABCA1-mediated cholesterol efflux. HeLa cells were incubated with U18666A and transfected as described above and cholesterol efflux was measured 24 hours later. Routine transfection efficiency is between 50–60%. Upon sustained ARF6 activation, cholesterol efflux increased by over 30% (Figure 1D). These results suggest that ARF6 activation reduces cholesterol accumulation by stimulating its movement to the plasma membrane and subsequent removal, likely via its well-established role in endosomal membrane recycling. In this way, stimulation of ARF6-mediated endosomal recycling may act as a compensatory mechanism to shift endosomal transport equilibrium in favor of recycling, in turn alleviating cholesterol accumulation in the LE/LY.

**Figure 2:**
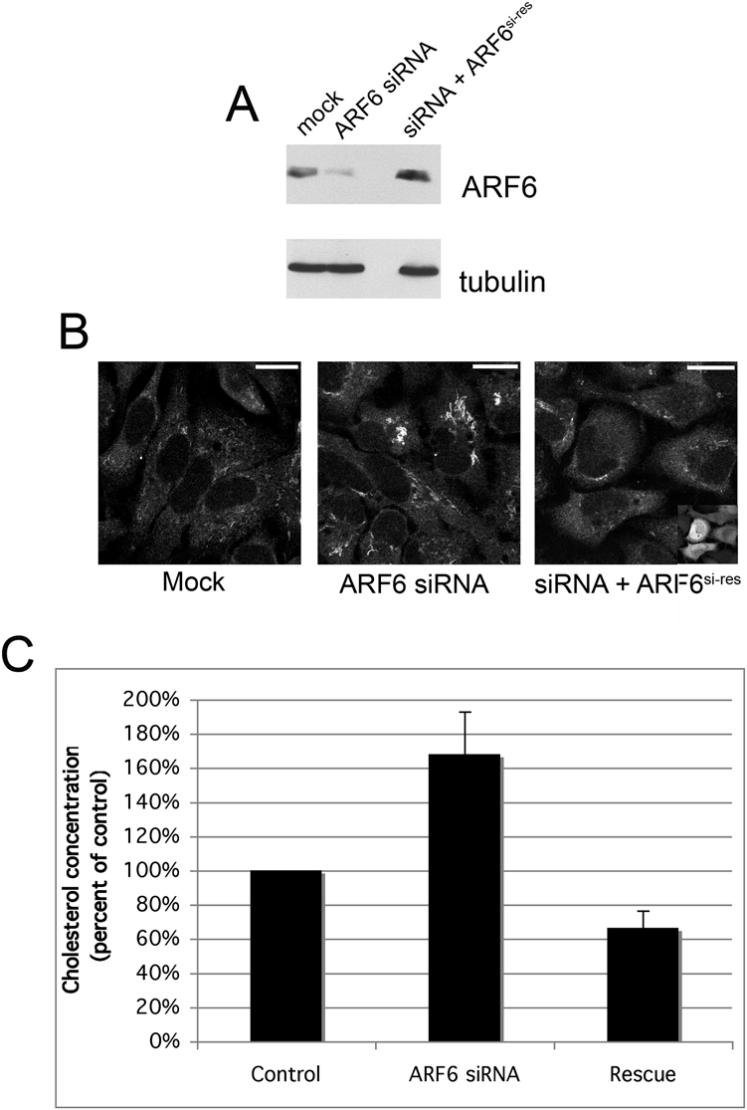
Depletion of ARF6 promotes cholesterol accumulation in normal cells. HeLa cells were mock-treated with transfection reagent alone or transfected with siRNA directed against ARF6 alone or plus pIRES-GFP-ARF6^si-res^ (“Rescue”) and analyzed 48 h later. *A*, ARF6 levels in cell lysates were determined using Western Blot analysis and probing for mouse anti-α-tubulin antibody (loading control) and a monoclonal antibody against ARF6. *B*, Cholesterol localization in fixed cells was assessed using BCθ toxin and streptavidin conjugated to Alexa Fluor 594. All three images were acquired in the same experiment using identical acquisition parameters. Contrast was enhanced using identical parameters in Adobe Photoshop 7.0. Inset in last image displays GFP expression indicating cells transfected with pIRES-GFP-ARF6^si-res^. Bars, 20 µm. *C*, Quantitation of cholesterol concentration using colorometric assay (see Methods). Values shown are percent of control. For each condition, the average of four independent experiments is shown with standard error bars. Statistically significant comparisons: ARF6 siRNA vs. control, p = 0.056 and ARF6 siRNA vs. Rescue, p = 0.012, using a two-tailed t-test.

Next, we investigated whether ARF6 is required for cholesterol homeostasis under normal conditions. We depleted ARF6 protein levels by transfecting cells with ARF6 siRNA and 48 h after transfection, we examined cholesterol distribution. As shown in Figure 2B, we found that siRNA-treated cells exhibited significant cholesterol accumulation inside the cell as visualized by staining with BCθ (biotinylated θ-toxin). Like filipin, BCθ binds to cholesterol-rich domains and is visualized using fluorescently labeled streptavidin [15]. Compared to filipin staining, use of BCθ leads to less labeling of the cell surface, less perturbation of membrane-lipid organization and more distinct labeling of internal cholesterol membranes [15]. When co-transfected with siRNA and a wild-type ARF6 construct bearing silent mutations in the region targeted by RNAi, no cholesterol accumulation was observed (Figure 2B, right panel). Using a biochemical assay to measure cholesterol levels, we found that ARF6-depleted cells exhibited greater than 65% increase in cholesterol after 48 h of siRNA treatment (Figure 2C). These results show that ARF6 is important for normal cholesterol homeostasis. These findings support the idea that perturbations in membrane recycling—in this case through the depletion of ARF6—can lead to cholesterol accumulation.

**Figure 3:**
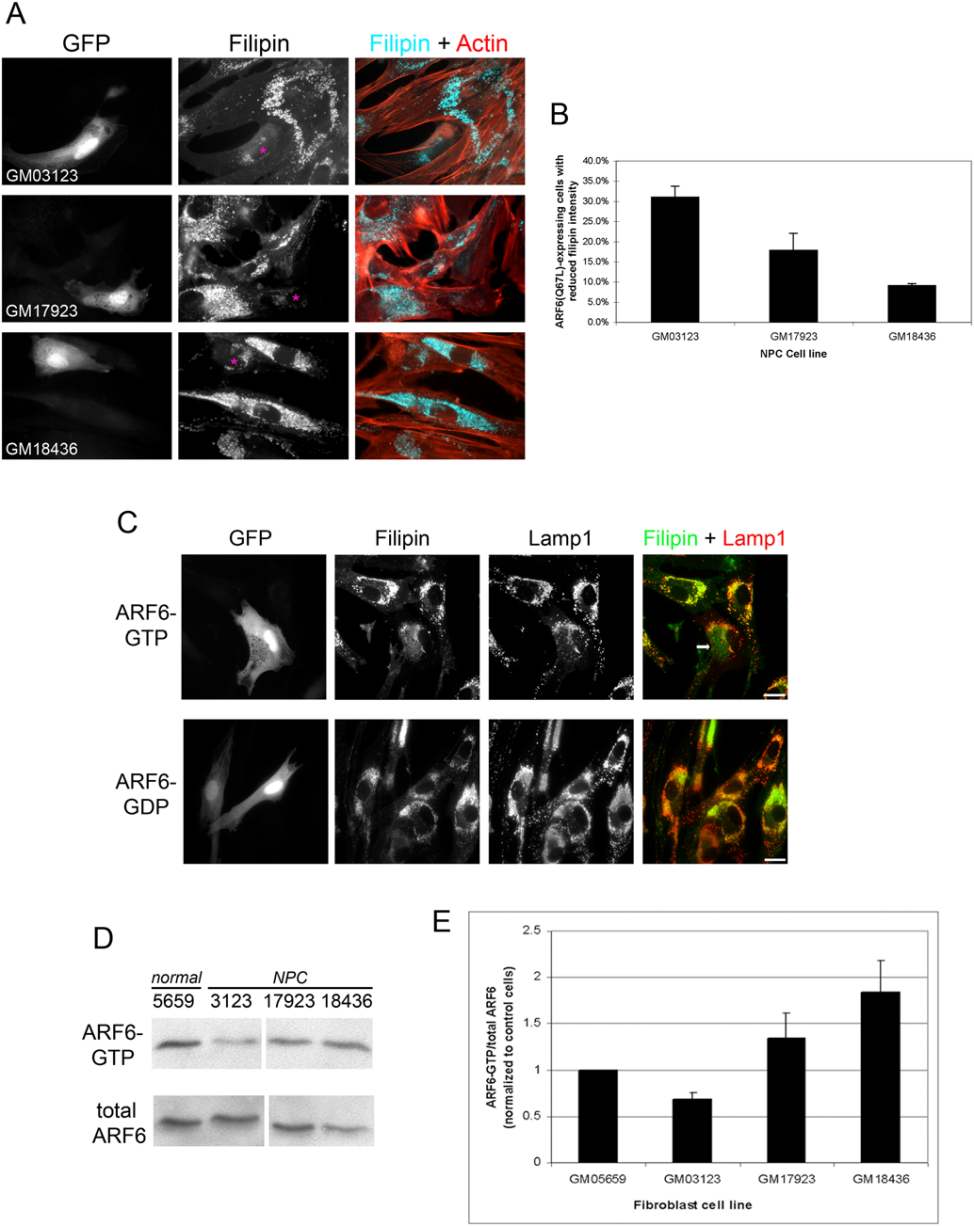
Reduction in cholesterol accumulation induced by ARF6-GTP expression is coupled to endogenous ARF6-GTP levels in NPC mutant fibroblasts. *A*, NPC mutant fibroblasts (GM03123, GM17923, and GM18436) were transfected with pIRES-GFP bearing ARF6(Q67L). Cells were fixed approximately 24 h post-transfection and stained with filipin (pseudo-colored blue in merged image) and counterstained with rhodamine phalloidin (red in merged image). Transfected cells are marked with asterisks in filipin images. Note decreased filipin staining in GFP-positive cells. *B*, NPC mutant fibroblasts (GM03123, GM17923 and GM18436) were treated as in *A*. Transfected cells with filipin intensity that was lower than neighboring non-transfected cells (as depicted in *A*) were scored (see Methods). 80–100 transfected cells of each cell line were counted in each of three independent experiments. The average percentage of transfected cells displaying reduced filipin intensity is graphed. Standard error bars are shown. Statistically significant comparisons: GM03123 vs. GM17923, p = 0.0117 and GM03123 vs. GM18436, p = 0.0114. *C*, GM03123 cells were transfected with pIRES-GFP bearing ARF6(Q67L) (top row) or ARF6(T27N) (bottom row). Cells were fixed approximately 24 h post-transfection and stained with filipin (pseudo-colored green in merged image) and immunolabeled for Lamp1 (red in merged image). Bars, 20 µm. Arrow points to region where filipin staining no longer overlaps with Lamp1. *D*, Lysates were prepared from normal (GM05659) and NPC mutant fibroblasts (GM03123, GM17923, GM18436) and subjected to the GST-MT-2 pull-down assay and probed for ARF6. Top row is ARF6 precipitated with GST-MT-2 beads and bottom row is ARF6 from the total cell lysate. *E*, Immunoblots from three independent experiments were subjected to densitometric analysis and the ARF6-GTP/total ARF6 ratios were calculated and normalized to control levels (GM05659). Standard error bars are shown. Statistically significant comparisons: GM03123 vs. GM05659, p = 0.011 and GM18436 vs. GM05659, p = 0.022.

Next we determined how sustained ARF6 activation impacts cholesterol accumulation observed in fibroblasts derived from NPC patients. Normal (GM05659) and NPC mutant fibroblasts (GM03123, GM17923, and GM18436) were transfected with the ARF6-GTP mutant, ARF6(Q67L). In each case, sustained ARF6 activation led to a reduction in filipin intensity (Figure 3A, top row, and Figure S2). Notably, the extent of response to ARF6-GTP expression varied among NPC fibroblasts derived from different patients (Figure 3B). In GM03123 cells, we observed reduced filipin intensity in 31% of transfected cells compared to neighboring, non-transfected cells. However, less than 18% of transfected GM17923 cells exhibited reduced filipin intensity and even fewer GM18436 cells responded to sustained ARF6 activation with a reduction in filipin intensity (9%; Figure 3B). In NPC cells, cholesterol-rich LE/LY compartments stain positive for lysosome markers, such as Lamp1. We observed that approximately 25% of the NPC cells expressing constitutively active ARF6 display a pool of filipin staining with significantly reduced overlap with Lamp1 (see arrows in Figure 3C and Figure S3), consistent with the hypothesis that sustained activation of ARF6 can lead to exit of cholesterol from the Lamp1-positive LE/LY compartments. We found no change in filipin intensity or overlap with Lamp1 in cells transfected with dominant negative ARF6, ARF6(T27N) (Figure 3C, bottom row). To investigate whether the varied response to ARF6 activation in NPC cells was coupled to endogenous ARF6-GTP levels, we examined the levels of endogenous ARF6-GTP using a GST-MT2 pull-down assay [16]. In each case, we compared the ratio of ARF6-GTP to total ARF6. Of the NPC fibroblasts we examined, GM03123 exhibited greater than 30% reduction in the level of ARF6-GTP compared to control cells, GM17923 had the same level as control cells, and GM18436 had a higher level of ARF6-GTP (Figure 3C,D). Thus, sustained ARF6 activation induced the most significant reduction in cholesterol accumulation in the NPC fibroblasts with the lowest endogenous levels of ARF6-GTP (GM03123).

**Figure 4:**
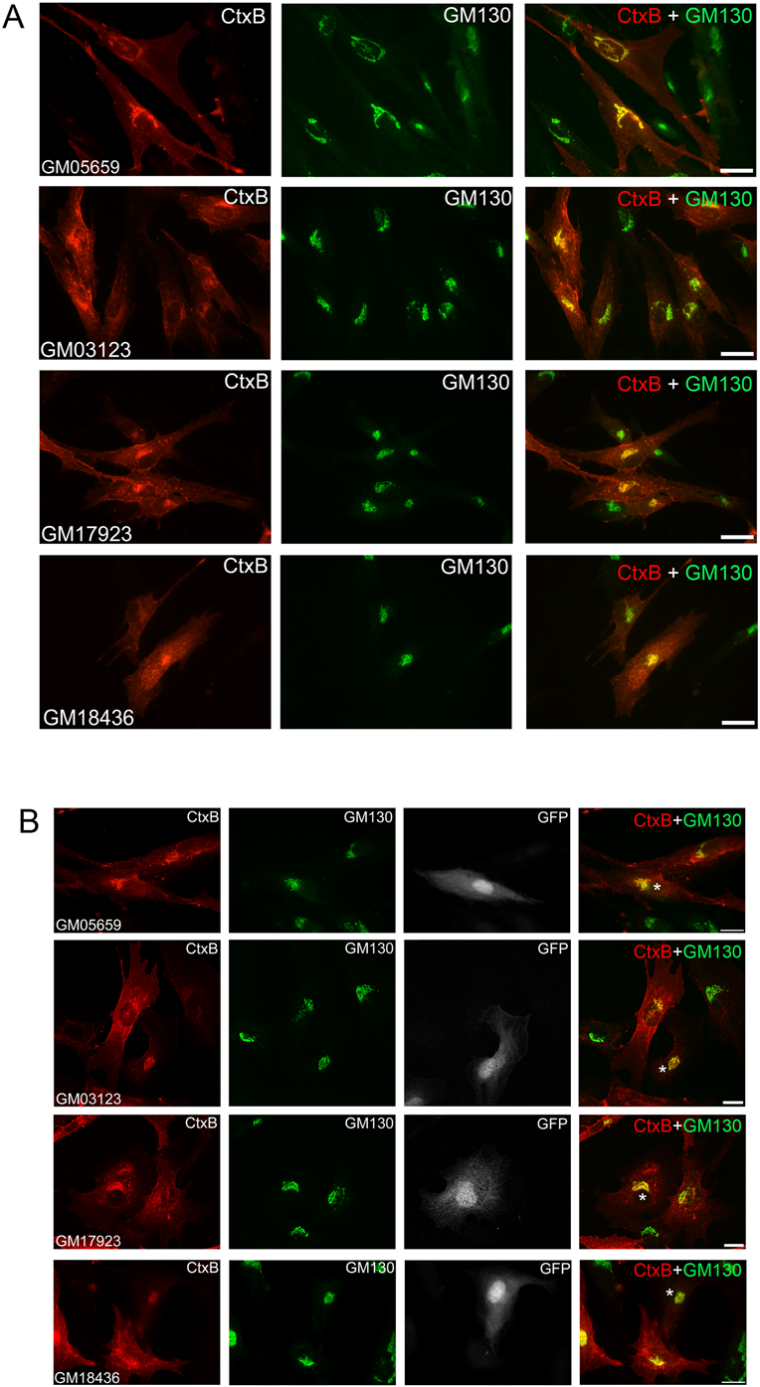
ARF6-GTP expression restores sphingolipid targeting to the Golgi. *A,* CtxB-AlexaFluor555 internalization in control (GM05659) and NPC mutant fibroblasts (GM03123, GM17923, and GM18436) was performed as described in Methods. To localize the Golgi, cells were fixed and immuno-labeled for GM130 with a mouse monoclonal antibody and a Cy5-conjugated anti-mouse secondary antibody (pseudo-colored green). *B*, Control and NPC mutant fibroblasts were transfected with ARF6(Q67L)-pIRES-GFP (monochrome, third row from left), subjected to CtxB-AlexaFluor555 internalization 20 h post-transfection, and immuno-labeled for GM130 (pseudo-colored green) as described in *A*. Transfected cells are marked with asterisks in merged images. See Table 1 for quantification of Golgi targeting of Ctx in NPC fibroblasts transfected with ARF6(Q67L)-pIRES-GFP.

We also investigated the impact of ARF6 activation on the distribution of other lipids, such as glycosphingolipids, that traffic inappropriately and accumulate with cholesterol in LE/LY compartments in NPC [14], [17]. We analyzed the distribution of cholera toxin (Ctx) that binds to GM1 ganglioside at the PM and serves as a marker for its localization. In normal cells, after internalization Ctx targets to the Golgi via retrograde traffic, whereas in NPC cells, Ctx accumulates instead in endosomal vesicles throughout the cytoplasm. Cholesterol depletion using methyl-β-cyclodextrin effectively restores normal Golgi targeting of Ctx [17]. Thus we determined if the reduction in cholesterol accumulation induced by sustained ARF6 activation is sufficient to restore Golgi targeting of internalized Ctx. As seen in Figure 4A, the efficiency of Ctx targeting to the Golgi in NPC fibroblasts was noticeably reduced as evidenced by increased labeling of Ctx in vesicles throughout the cytoplasm and in marked contrast with control cells that showed efficient targeting of Ctx to the Golgi. Next, we examined the effect of exogenous ARF6-GTP on Ctx trafficking in NPC fibroblasts. We observed increased targeting of Ctx to the Golgi, as evidenced by reduced Ctx label outside of the Golgi (Figure 4B). We quantitated the percentage of Ctx label at the Golgi compared to the total Ctx label in transfected and non-transfected NPC fibroblasts. We found expression of constitutively active ARF6 increased targeting of Ctx to the Golgi by 80–90% (see Table 1). NPC cells expressing dominant negative ARF6, ARF6(T27N), exhibited no change in Ctx targeting (Figure S4). These findings suggest that the reduction in cholesterol storage induced by sustained ARF6 activation allows for restoration of normal lipid trafficking. Alternatively, ARF6 itself may be involved in the trafficking of Ctx to the Golgi and expression of ARF6-GTP may stimulate its transport to the Golgi.

**Table 1:** Golgi targeting of internalized Ctx in NPC mutant fibroblasts. NPC fibroblasts were transfected with ARF6(Q67L)-pIRES-GFP and Ctx internalization was analyzed (as in Figure 4B). The percentage of Ctx localized to the Golgi, as described in Methods, is shown here.

NPC Fibroblast Line	Percentage of Ctx label in the Golgi (average percent±SD)		Percent Increase in Golgi targeting of Ctx
	Non-transfected	ARF6(Q67L)-transfected	
GM03123	8.3±1.4	15.1±4.6	81%
GM17923	9.3±1.9	18.0±4.6	94%
GM18436	9.7±2.2	18.5±6.7	91%

Although lipid accumulation is a defining characteristic of NPC cells, defects in the recycling of protein, lipid, and soluble ligands accompany the NPC phenotype [8], [9], [17], [18]. In addition to a potential role for NPC1, an underlying cause for trafficking defects in NPC may be changes in the activity of proteins that regulate endosomal membrane trafficking. For example, Rab4, an early endosomal Rab GTPase that facilitates endosomal recycling, is expressed at higher levels in NPC cells, but its activity is compromised by a decrease in its ability to be extracted from endosomal membranes [18]. In the present study we find that levels of activated ARF6 are suppressed in fibroblasts derived from an NPC patient. Thus, an inhibition of endosomal recycling due to changes in the activity of regulatory proteins, such as ARF6, may contribute directly to the NPC phenotype by promoting cholesterol accumulation. This work is consistent with other reports showing that Rab proteins involved in endosome membrane cycling, such as Rab11 and Rab8, also impact normal cholesterol dynamics [19], [20].

Previous studies have shown that late endocytic traffic, mediated by Rab7 and Rab9, is compromised in NPC cells [9], [21], [22]. Overexpression of Rab 7 and 9 proteins reduced the NPC phenotype in cultured cells and in mice, suggesting that stimulation of traffic out of the late endosome and lysosome may bypass the block caused by NPC1 mutations [23]–[26]. Our findings support the involvement of endosomal recycling in cholesterol accumulation and identify that stimulation of endosomal recycling reverses the NPC phenotype.

Finally, our results also demonstrate that cell lines derived from different NPC patients display different relative levels of endogenous ARF6-GTP. Furthermore, the reduction in cholesterol accumulation we observe in response to sustained ARF6 activation varies inversely with the relative level of endogenous ARF6-GTP. Thus, ARF6 activation compensates for the NPC defect most effectively in the fibroblasts with the lowest levels of endogenous ARF6-GTP. These findings suggest that changes in membrane trafficking pathways or activities of associated regulatory proteins may contribute differently to individual cases of NPC. Such variances among NPC cases should therefore be taken into account when formulating therapeutic strategies aimed at modulating the trafficking of cholesterol or other sphingolipids.

## Methods

### Reagents

Filipin III was obtained from Sigma or Cayman Chemical. Rhodamine phalloidin, streptavidin conjugated to Alexa Fluor 594, cholera toxin subunit B (Ctx) conjugated to Alexa Fluor® 555 and anti-mouse secondary antibody conjugated to Cy5 were from Invitrogen. Apolipoprotein AI and rabbit polyclonal antibody against the hemagglutinin (HA) tag were from Sigma Chemical, U18666A from Calbiochem and [1,2-^3^H-(N)]-cholesterol from Perkin Elmer. BCθ toxin was provided by Yoshiko Ohno-Iwashita (Tokyo, Japan). Mouse monoclonal antibody against GM130 was from BD Biosciences Pharmingen.

### Cell culture and plasmid transfection

HeLa cells were cultured in Dulbecco's modified Eagle's medium supplemented with 10% fetal bovine serum, 2 mM L-glutamine, penicillin, and streptomycin (complete DMEM). Three primary human skin fibroblast lines derived from NPC1 patients (GM03123, GM18436, GM17923) and one primary human skin fibroblast line from an unaffected patient (GM05659) were obtained from Coriell Cell Repositories (Camden, NJ). They were cultured in Minimum Essential Medium with Earle's salts (Mediatech) with 2 mM L-glutamine, 10% fetal bovine serum, penicillin, and streptomycin. pcDNA3.1 bearing HA-tagged ARF6 and pIRES-EGFP bearing ARF6(T27N) or ARF6(Q67L) have been previously described [16], [27]. Transient plasmid transfections were performed using FuGENE transfection reagent (Roche Molecular Biochemicals), following the manufacturer's instructions.

### Filipin fluorescence and BCθ-toxin staining

For filipin staining, cells were fixed with 2%PFA for 30 min, washed with 1XPBS, and incubated with freshly prepared filipin solution (∼200 µg/ml in PBS containing 10% fetal bovine serum) for 30 minutes. Then, cells were washed with PBS and mounted in ProLong Anti-fade mounting media (Invitrogen) or processed for immunofluorescence. Cells were observed on an inverted microscope (Eclipse TE2000-U; Nikon) fitted for immunofluorescence with a 60× immersion lens and HighQ filter sets from Chroma (Brattleboro, VT). Images were acquired and analyzed using Metamorph 6.2r4 (Universal Imaging Corp.) and a Cascade 512B camera (Photometrics). To measure filipin intensity, regions of interest were drawn around each cell and the total pixel intensity for each cell was recorded using ImageJ. 40–60 cells were analyzed per experiment and three independent repetitions were performed for each transfection. We scored a cell with reduced filipin intensity if it displayed more than 30% reduction in filipin intensity compared with the average filipin intensity of untransfected cells from the same experiment. For BCθ labeling, cells were fixed with 4% PFA and stained with BCθ and Alexa594-conjugated streptavidin as described [15]. Cells labeled with BCθ were imaged on a Bio-Rad MRC1024 confocal system equipped with a Nikon Diaphot 200 fluorescence microscope. Images were adjusted for contrast using identical parameters in Adobe Photoshop, version 7.0.

### Cholesterol efflux

HeLa cells were seeded in a 12-well dish at 60% confluency. The next day cells were incubated with serum-free DMEM containing ^3^H-cholesterol (1 µCi/ml) and 0.2% BSA for 17 h. Then, U18666A (1 µg/ml) was added to the cells and they were incubated for another 7 h. Next, cells were transfected with the appropriate plasmid. After 2 h, transfection complexes were removed, and cells were incubated with fresh complete DMEM containing ^3^H-cholesterol (1 µCi/ml), 0.2% BSA and U18666A for 4–6 hours. Then, media was removed and cells were washed extensively with 1XPBS. To monitor efflux, cells were incubated with serum-free DMEM supplemented with apoA-I (10 µg/ml) +0.2%BSA and U18666A for 14 h, after which time media supernatant was collected and centrifuged for 5 min at 14,000 rpm to remove cell debris. Cells were lysed with 0.2N NaOH. The radioactivity in each fraction (media and cell lysate) was determined using a Beckman scintillation counter. Percent efflux was calculated as follows: (media cpm)/(cell lysate cpm + media cpm). Each experiment was carried out in triplicate and three independent repetitions were performed. The average efflux percents were compared using a two-tailed student's t-test (see Figure 1E).

### RNA interference and rescue

Endogenous levels of ARF6 were knocked-down in HeLa cells using siRNA oligonucleotides [28]. To create an siRNA-resistant ARF6 construct (for “rescue” experiments), pIRES-GFP bearing a wild-type copy of ARF6 was used for site-directed mutagenesis using the Stratagene QuikChange II kit. Mutagenesis introduced three silent mutations in ARF6 at the following nucleotide positions: A84C, C87A, G90A. Successful clones were confirmed by DNA sequencing. Fourty-eight hours after transfection, approximately 1×10^6^ cells were trypsinized and a small portion was used to make a protein lysate for Western blotting analysis. The remaining cells were used for lipid isolation and quantitation of cholesterol concentration using the colorometric Cholesterol/Cholesteryl Ester Quantitation Kit (Calbiochem). For this analysis, no esterase was added to the assay and the absorbance was read at 570 nm.

### Determination of relative ARF6-GTP levels

Cell lysates were prepared, subjected to the MT-2 pull-down assay, and analyzed by immunoblotting as described previously [16], using approximate 450 µg of protein lysate per assay. Western blots were scanned with an UltraScan XL laser Densitometer (Amersham Biosciences) and ARF6 precipitated with MT-2 was normalized to total ARF6 levels in the lysate. In each experiment, the level of ARF6-GTP in control cells (GM05659) was set to 1 and the levels in each NPC cell line relative to control is reported under “Results” (Figure 2D). The averages from three independent experiments were compared using a two-tailed student's t-test (see Figure 3D).

### Cholera toxin trafficking

Cells were incubated with 1.5 µg/ml CtxB-Alexa Fluor 555 in PBS for 30 minutes at 4°C, washed three times with PBS, and incubated in serum-free media for 1 hour at 37°C prior to fixation. To localize the Golgi, cells were labeled subsequently for GM130 using a mouse monoclonal antibody and Cy5-conjugated secondary antibody. Immunofluorescence techniques have been described previously [29]. To determine the percentage of Ctx localized to the Golgi, individual cells were examined using ImageJ and the total pixel intensity of the Ctx label within the Golgi was divided by the total pixel of the Ctx label in the entire cell. Results are presented in Table 1.

## Supporting Information

Figure S1 Cholesterol overlaps with ARF6 in HeLa cells and sustained activation of ARF6 decreases cholesterol accumulation. A, HeLa cells were transfected with HA-tagged ARF6(wt), fixed and stained with filipin (top right; pseudo-colored green in merged image) and immunolabeled for HA-tagged ARF6 (top left; red in merged image). Bottom right is an enlarged region of the merged image outlined with a white rectangle. Bar, 20 µm. B, HeLa cells were treated with U18666A to induce cholesterol accumulation and then transfected with pIRES-GFP bearing ARF6(Q67L), fixed approximately 18 h post-transfection, and stained with filipin (red in merged image). Transfected cells are marked with asterisks in filipin images. Note decreased filipin staining in GFP-positive cells.
(3.15 MB TIF)

Figure S2 ARF6-GTP expression reduces cholesterol accumulation in NPC fibroblasts. NPC mutant fibroblasts (GM03123, GM17923 and GM18436) were transfected with pIRES-GFP bearing ARF6(Q67L) (left panel). Cells were fixed approximately 24 h post-transfection and stained with filipin (right panel). Transfected cells are marked with asterisks in filipin images. Bars, 20 µm.
(1.85 MB TIF)

Figure S3 NPC fibroblasts expressing ARF6-GTP display regions of filipin staining that are Lamp1-negative. NPC mutant fibroblasts (GM03123-left column and GM17923-right column) were transfected with pIRES-GFP-ARF6(Q67L). Cells were fixed approximately 24 h post-transfection and stained with filipin (pseudo-colored green in merged image) and immunolabeled for Lamp1 (red in merged image). Arrows point to areas of filipin staining that no longer overlap with Lamp1.
(3.12 MB TIF)

Figure S4 ARF6-GDP expression in control and NPC mutant fibroblasts does not affect CtxB traffic to the Golgi. Control (GM05659, top row) and NPC mutant fibroblasts (GM03123, bottom row) were transfected with ARF6(T27N)-pIRES-GFP (monochrome, third row from left) and subjected to CtxB-AlexaFluor555 internalization 20 h post-transfection as described in Methods. Cells were fixed and immunolabeled for GM130 with a mouse monoclonal antibody and a Cy5-conjugated anti-mouse secondary antibody (pseudo-colored green in merged image). Transfected cells are marked with asterisks in merged images. Bar, 20 µm
(2.48 MB TIF)
