# Supplementary figures and images for: ARF6-Mediated Endosome Recycling Reverses Lipid Accumulation Defects in Niemann-Pick Type C Disease
Source: PLoS One. 2009 Apr 14;4(4):e5193. doi: 10.1371/journal.pone.0005193 (PMC2664925; doi:10.1371/journal.pone.0005193)

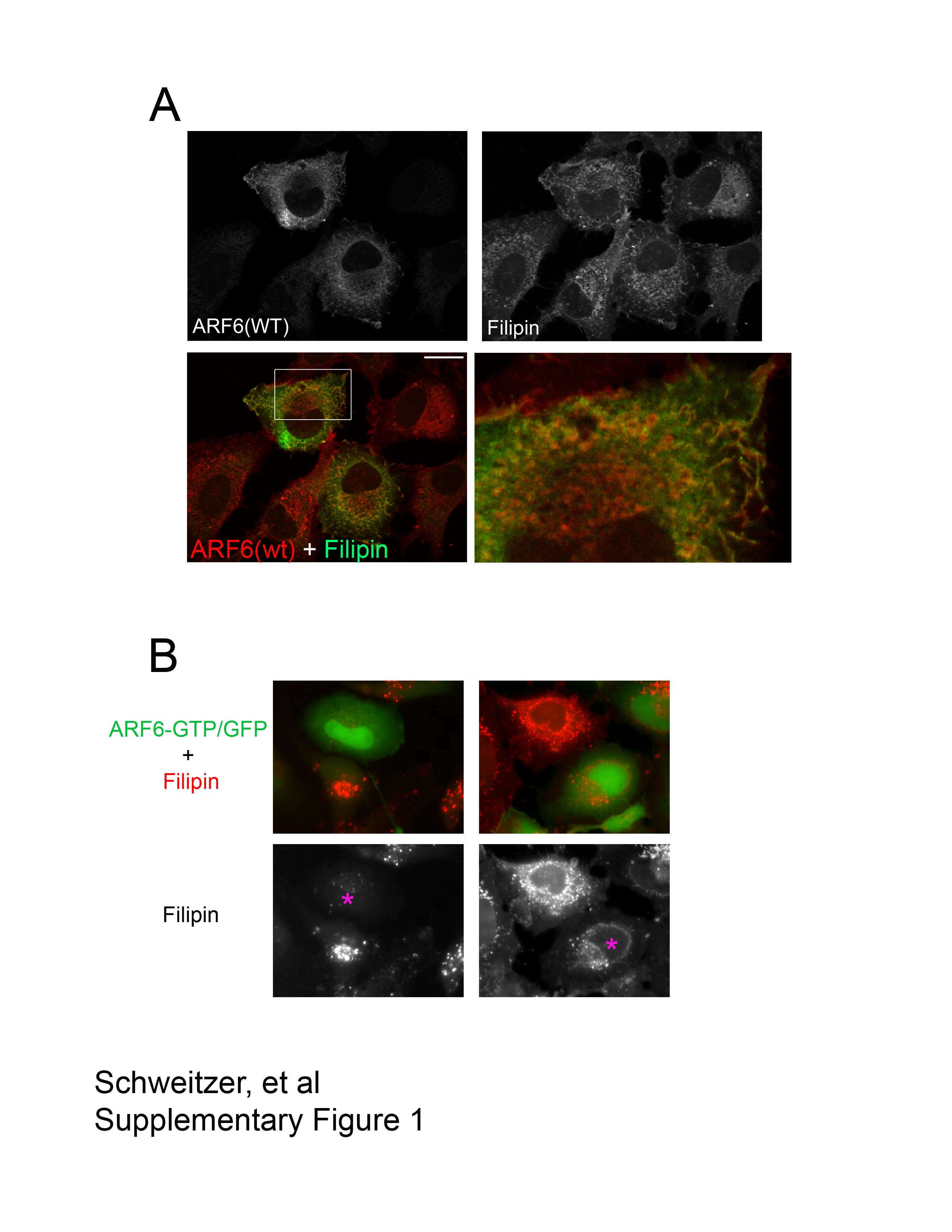

Supplement: Figure S1 — Cholesterol overlaps with ARF6 in HeLa cells and sustained activation of ARF6 decreases cholesterol accumulation. A, HeLa cells were transfected with HA-tagged ARF6(wt), fixed and stained with filipin (top right; pseudo-colored green in merged image) and immunolabeled for HA-tagged ARF6 (top left; red in merged image). Bottom right is an enlarged region of the merged image outlined with a white rectangle. Bar, 20 µm. B, HeLa cells were treated with U18666A to induce cholesterol accumulation and then transfected with pIRES-GFP bearing ARF6(Q67L), fixed approximately 18 h post-transfection, and stained with filipin (red in merged image). Transfected cells are marked with asterisks in filipin images. Note decreased filipin staining in GFP-positive cells. (3.15 MB TIF) [file pone.0005193.s001.tif]

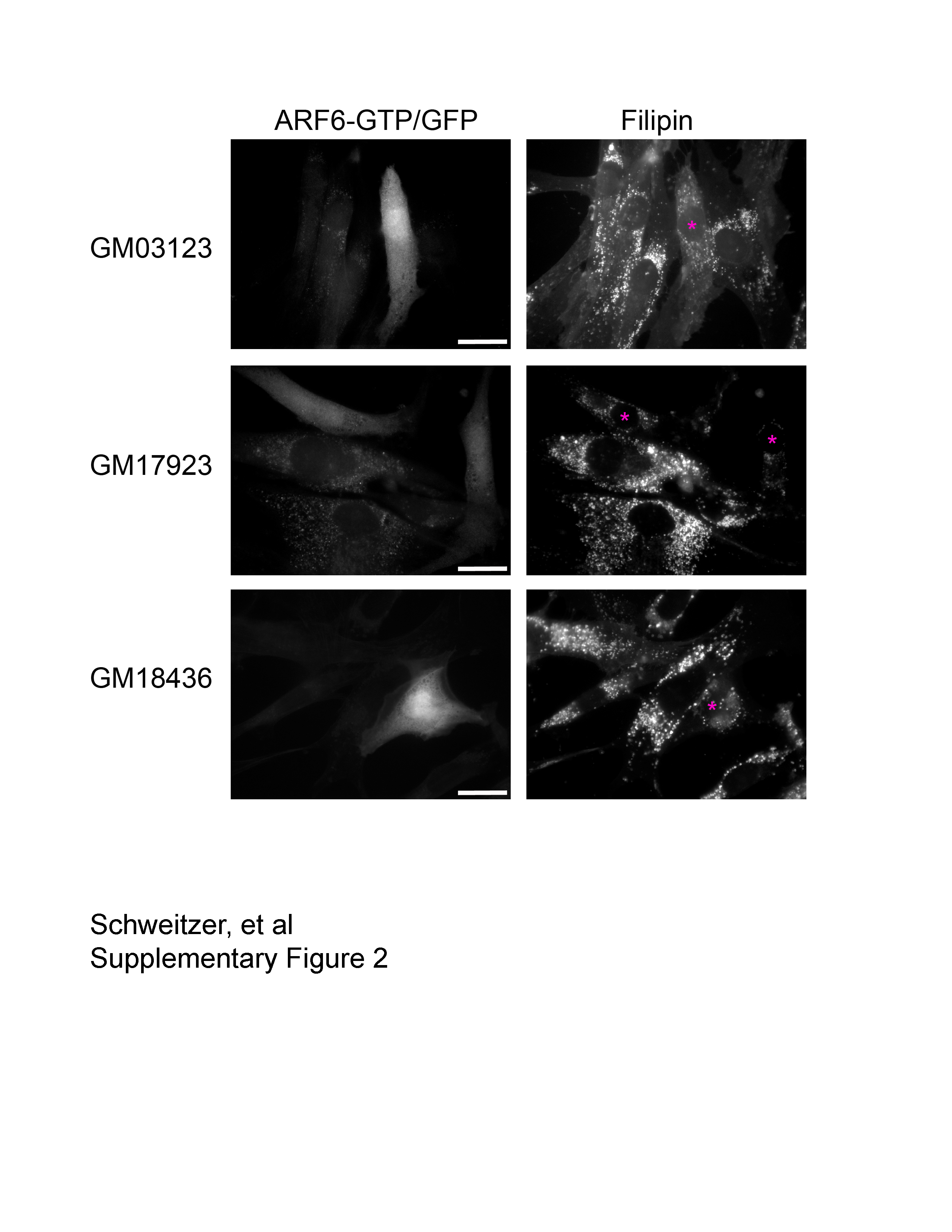

Supplement: Figure S2 — ARF6-GTP expression reduces cholesterol accumulation in NPC fibroblasts. NPC mutant fibroblasts (GM03123, GM17923 and GM18436) were transfected with pIRES-GFP bearing ARF6(Q67L) (left panel). Cells were fixed approximately 24 h post-transfection and stained with filipin (right panel). Transfected cells are marked with asterisks in filipin images. Bars, 20 µm. (1.85 MB TIF) [file pone.0005193.s002.tif]

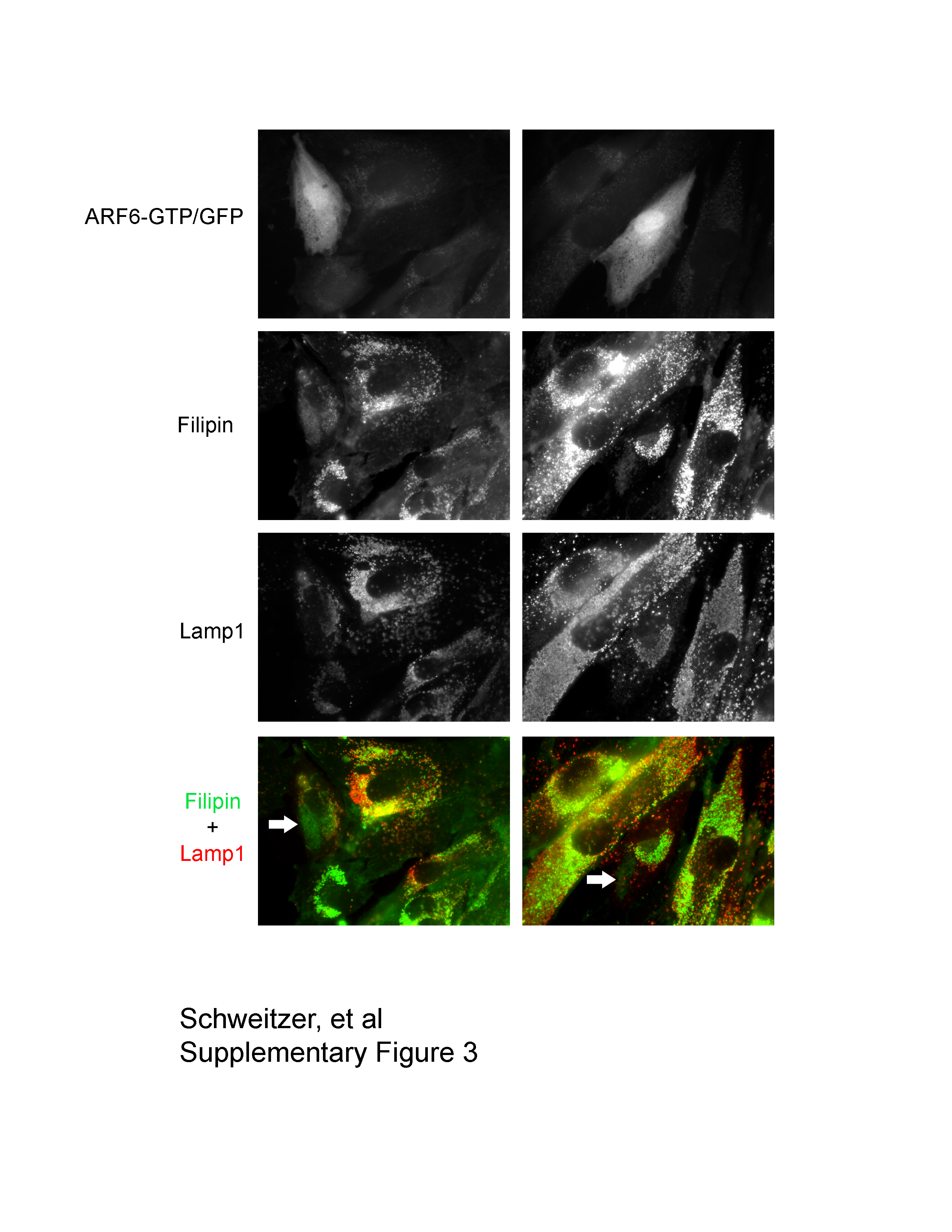

Supplement: Figure S3 — NPC fibroblasts expressing ARF6-GTP display regions of filipin staining that are Lamp1-negative. NPC mutant fibroblasts (GM03123-left column and GM17923-right column) were transfected with pIRES-GFP-ARF6(Q67L). Cells were fixed approximately 24 h post-transfection and stained with filipin (pseudo-colored green in merged image) and immunolabeled for Lamp1 (red in merged image). Arrows point to areas of filipin staining that no longer overlap with Lamp1. (3.12 MB TIF) [file pone.0005193.s003.tif]

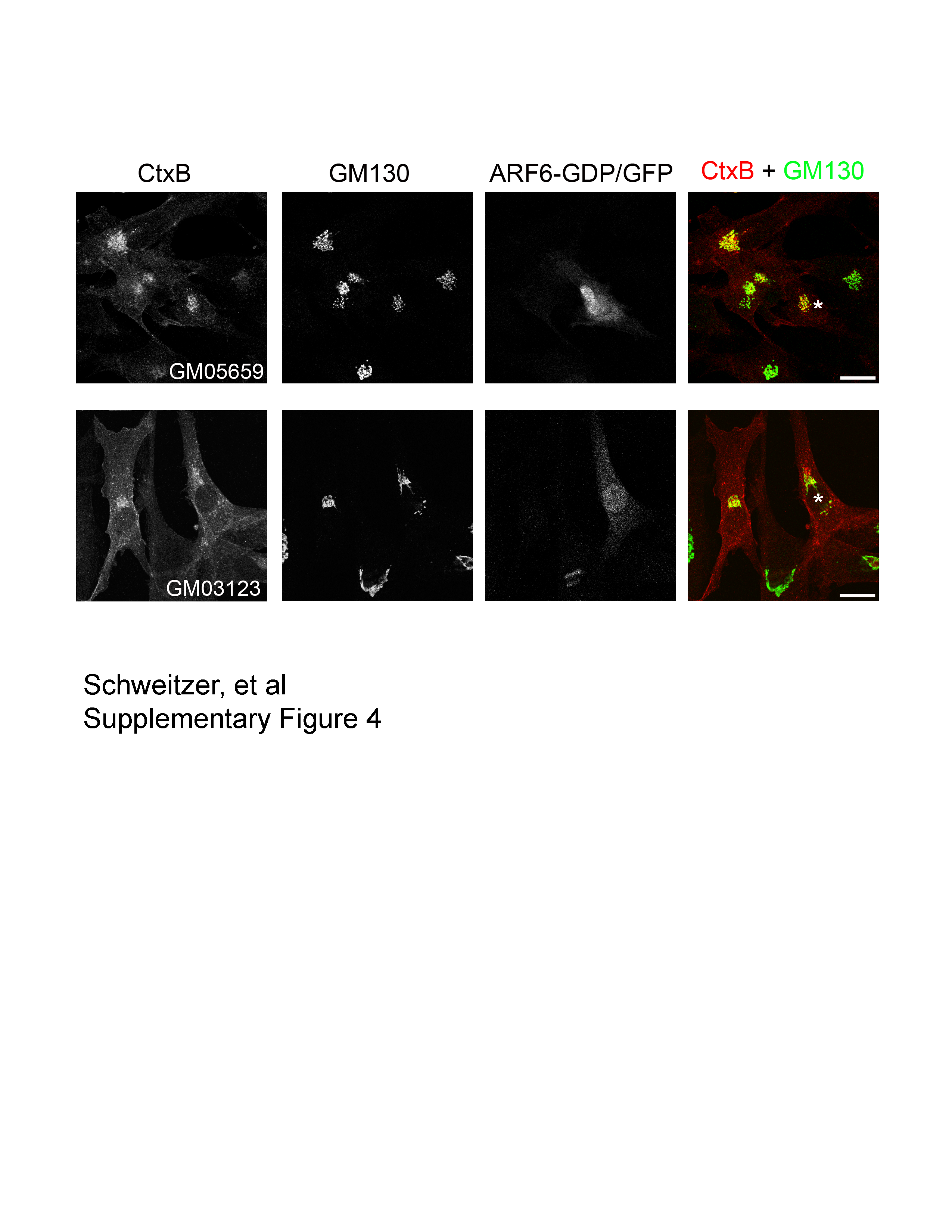

Supplement: Figure S4 — ARF6-GDP expression in control and NPC mutant fibroblasts does not affect CtxB traffic to the Golgi. Control (GM05659, top row) and NPC mutant fibroblasts (GM03123, bottom row) were transfected with ARF6(T27N)-pIRES-GFP (monochrome, third row from left) and subjected to CtxB-AlexaFluor555 internalization 20 h post-transfection as described in Methods. Cells were fixed and immunolabeled for GM130 with a mouse monoclonal antibody and a Cy5-conjugated anti-mouse secondary antibody (pseudo-colored green in merged image). Transfected cells are marked with asterisks in merged images. Bar, 20 µm (2.48 MB TIF) [file pone.0005193.s004.tif]
